# Effects of Transcranial Direct Current Stimulation Block Remifentanil-Induced Hyperalgesia: A Randomized, Double-Blind Clinical Trial

**DOI:** 10.3389/fphar.2018.00094

**Published:** 2018-02-19

**Authors:** Gilberto Braulio, Savio C. Passos, Fabricio Leite, Andre Schwertner, Luciana C. Stefani, Ana C. S. Palmer, Iraci L. S. Torres, Felipe Fregni, Wolnei Caumo

**Affiliations:** ^1^Post-graduate Program in Medical Sciences, Faculdade de Medicina da Universidade Federal do Rio Grande do Sul (UFRGS), Porto Alegre, Brazil; ^2^Pain and Palliative Care Service and Laboratory of Pain and Neuromodulation at HCPA, Hospital de Clínicas de Porto Alegre, Porto Alegre, Brazil; ^3^Department of Surgery Pain and Anesthesia, School of Medicine, Faculdade de Medicina da Universidade Federal do Rio Grande do Sul (UFRGS), Porto Alegre, Brazil; ^4^Post-graduate Program in Pharmacology and Therapeutic, Instituto de Ciências Básicas da Saúde, Universidade Federal do Rio Grande do Sul, Porto Alegre, Brazil; ^5^Department of Pharmacology, Instituto de Ciências Básicas da Saúde, Universidade Federal do Rio Grande do Sul, Porto Alegre, Brazil; ^6^Department of Physical Medicine and Rehabilitation, Harvard Medical School, Boston, MA, United States; ^7^Department of Neurology, Harvard Medical School, Boston, MA, United States; ^8^Berenson-Allen Center for Noninvasive Brain Stimulation, Boston, MA, United States; ^9^Department of Neurology, Beth Israel Deaconess Medical Center, Harvard Medical School, Boston, MA, United States

**Keywords:** tDCS, hyperalgesia, remifentanil, pain threshold, CPM

## Abstract

**Background:** Remifentanil-induced hyperalgesia (r-IH) involves an imbalance in the inhibitory and excitatory systems. As the transcranial Direct Current Stimulation (tDCS) modulates the thalamocortical synapses in a top-down manner, we hypothesized that the active (a)-t-DCS would be more effective than sham(s)-tDCS to prevent r-IH. We used an experimental paradigm to induce temporal summation of pain utilizing a repetitive cold test (rCOLDT) assessed by the Numerical Pain Score (NPS 0-10) and we evaluated the function of the descending pain modulatory system (DPMS) by the change on the NPS (0–10) during the conditioned pain modulation (CPM)-task (primary outcomes). We tested whether a-tDCS would be more effective than s-tDCS to improve pain perception assessed by the heat pain threshold (HPT) and the reaction time during the ice-water pain test (IPT) (secondary outcomes).

**Methods:** This double-blinded, factorial randomized trial included 48 healthy males, ages ranging 19–40 years. They were randomized into four equal groups: a-tDCS/saline, s-tDCS/saline, a-tDCS/remifentanil and s-tDCS/remifentanil. tDCS was applied over the primary motor cortex, during 20 min at 2 mA, which was introduced 10 min after starting remifentanil infusion at 0.06 μg⋅kg^-1^⋅min^-1^ or saline.

**Results:** An ANCOVA mixed model revealed that during the rCOLDT, there was a significant main effect on the NPS scores (*F* = 3.81; *P* = 0.01). The s-tDCS/remifentanil group presented larger pain scores during rCOLDT, [mean (*SD*) 5.49 (1.04)] and a-tDCS/remifentanil group had relative lower pain scores [4.15 (1.62)]; showing its blocking effect on r-IH. a-tDCS/saline and s-tDCS/saline groups showed lowest pain scores during rCOLDT, [3.11 (1.2)] and [3.15 (1.62)], respectively. The effect of sedation induced by remifentanil during the rCOLDT was not significant (*F* = 0.76; *P* = 0.38). Remifentanil groups showed positive scores in the NPS (0–10) during the CPM-task, that is, it produced a disengagement of the DPMS. Also, s-tDCS/Remifentanil compared to a-tDCS showed lower HPT and larger reaction-time during the IPT.

**Conclusion:** These findings suggest that effects of a-tDCS prevent the summation response induced by r-IH during rCOLDT and the a-tDCS blocked the disengagement of DPMS. Thereby, tDCS could be considered as a new approach to contra-regulate paradoxical mechanisms involved in the r-IH. Clinical trials identification: NCT02432677. URL:https://clinicaltrials.gov/.

## Introduction

Opioids are the most effective analgesics to treat moderate to severe acute and chronic pain. However, growing evidence shows that opioids can elicit unexpected changes in pain sensitivity. This hyperalgesia induced by opioids (OIH) may extend beyond the postoperative period and can lead to the development of chronic pain persisting for months (Salengros et al., 2010). Considering that OIH is a paradoxical response, a better comprehension about its mechanism could help the clinician plan preventive approaches to reduce acute postoperative pain, and possibly to reduce the incidence of persistent post-surgical pain potentially related to the OIH, which ranges 16–70% (Salengros et al., 2010). A recent systematic review that involved 27 studies and 1494 patients add evidence of OIH in humans (Fletcher and Martinez, 2014), which cause a significant increase in postoperative pain intensity at rest persisting 24 h after surgery. Another study found that patients undergoing thoracotomy who received high-dose remifentanil without epidural analgesia experienced a three times larger area of allodynia compared to low-dose infusion of remifentanil (Salengros et al., 2010). At 6 months followed-up, a higher incidence of chronic pain resembling neuropathic pain was observed in those receiving high doses of remifentanil (Salengros et al., 2010). Although there is a mixed result, pre-clinical and clinical evidence has demonstrated that remifentanil might induce hyperalgesia (r-IH) either with acute or chronic use (Kim et al., 2014; Stoicea et al., 2015; Yu et al., 2016). The most reliable proof of opioids producing hyperalgesia (OIH) in humans comes from opioid infusions in healthy volunteers (Fishbain et al., 2009).

According to previous reports, morphine blocks the diffuse noxious inhibitory control (DNIC) effects in rats (Bouhassira et al., 1992, 1993) and healthy humans (Le Bars et al., 1992) in a naloxone-reversible fashion (Le Bars et al., 1981; Willer et al., 1990). Another study showed that patients with chronic pain treated with oral opioids exhibited less capacity to inhibit pain signals compared to non-treated patients (Chu et al., 2006). Specifically related to remifentanil, an attractive hypothesis is that its effects involve the *N*-methyl-D-aspartate (NMDA) receptors (NR1A/2A and NR1A/2B), and hence, it induces a dysfunction of the descending pain modulatory system (DPMS), which is rich in both inhibitory mu-opioid and excitatory NMDA receptors (Hahnenkamp et al., 2004). In fact, in one experimental study in humans, S-ketamine abolished the r-IH (Joly et al., 2005). Also, knockout mice without μ-, δ-, or κ-opioid receptors develop thermal hyperalgesia when exposed to acute or chronic fentanyl use (Celerier et al., 2001). Finally, when morphine-6β-glucuronide (a metabolite with μ-receptor agonist activity) was administered concurrently with the opioid receptor antagonist naloxone, it leads to OIH by an opioid receptor-independent mechanism (Van Dorp et al., 2009). Despite the growing evidence of OIH, the results are heterogeneous, thus revealing a persistent gap to study whether top-down modulatory approaches can improve the inhibitory function of the descending corticospinal pathways since their dysfunction mediate this paradoxical effect.

As aforementioned, OIH involves a dysfunction in the thalamus cortical pathways as well as in the DPMS. Thereby, transcranial direct current stimulation (tDCS) is a promising approach to contra-regulate OIH, because it modulates the thalamocortical synapses in a top-down manner within pain pathways (Stagg et al., 2009). The tDCS effect depends on the polarity, positioning, and size of electrodes, as well as the duration and intensity of the current flow (DaSilva et al., 2011). It has been effective either in acute postoperative pain (Ribeiro et al., 2017) and chronic pain (i.e., trigeminal neuralgia, phantom pain, fibromyalgia, etc.) (Fregni et al., 2006; Bolognini et al., 2013). Although its effect on pain is not completely understood, it downregulates the emotional component of the pain experience while alleviating pain via activation of the descending pain suppression system (Hadjipavlou et al., 2006). Indeed, the majority of trials has rationalized that the tDCS effect involves changes in “brain function” induced by excitatory or inhibitory boosts. According to studies of rodent brain slices *in vitro*, tDCS can affect long-term-potentiation (LTP) dependent on *N*-methyl D-aspartate (Giordano et al., 2017). Also, its neuroplastic changes involves a regulation of a broad variety of different interneurons and neurotransmitters, such as opioidergic (Nitsche et al., 2003; Filmer et al., 2014), GABAergic (Nitsche et al., 2004), glutaminergic (Nitsche et al., 2003), cholinergic (Kuo et al., 2007), serotonergic (Nitsche et al., 2009), and dopaminergic (Nitsche et al., 2006).

In this sense, to investigate the therapeutic effect of tDCS in OIH, we used accurate tests to evoke OIH, as well appropriate approaches to detect and measure its presence. According to previous studies, OIH is dependent on the nature of the pain model used, and the cold pain test has demonstrated to be the most sensitive of the methods tested in detecting opioid-related hyperalgesia (Krishnan et al., 2012). Thus, due to the complex mechanisms of pain, innovative interventions should be tested in an experimental paradigm that allows us to characterize the etiological components of pain (nature, localization, intensity, frequency, and duration of the trigger to evoke pain). Hence, stimuli to elicit pain should be suitable to activate the pain pathways, while it being non-invasive and permit its repeated application (Olesen et al., 2012).

Taking this into account, and considering how tDCS modulates the thalamocortical synapses in a top-down manner, we hypothesized that the active (a)-t-DCS would be more effective than a sham(s)-tDCS to prevent r-IH. We used an experimental paradigm to induce temporal summation of pain utilizing a repetitive cold test (rCOLDT) assessed by the Numerical Pain Score (NPS 0-10) and we evaluated the function of the DPMS by the change on the NPS (0–10) during the conditioned pain modulation (CPM)-task (primary outcomes). We tested whether a-tDCS would be more effective than s-tDCS to improve pain perception assessed by the heat pain threshold (HPT) and the reaction time during the ice-water pain test (IPT) (secondary outcomes).

## Materials and Methods

### Design Overview, Settings, and Participants

**FIGURE 1:**
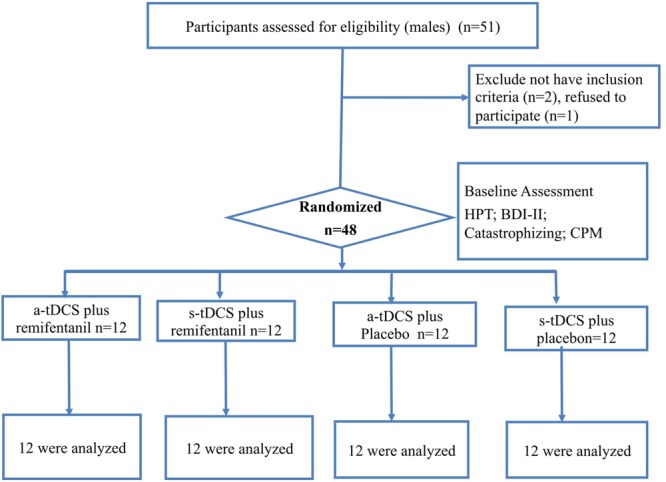
Randomization and follow-up of the study subject.

This is a randomized, double-blinded, parallel clinical trial, conducted by the CONSORT guidelines. This study was registered at the ClinicalTrials.gov on April 6, 2015 (NCT02432677, investigator WC.). The Research Ethics Committee at the Hospital de Clinicas de Porto Alegre approved this protocol. All volunteers provided their written informed consent before participating in the study. The Experimental design, assessments, and interventions are presented in **Figure 1**.

### Study Subjects

The volunteers were recruited from the general population by advertisement postings in public places in the Porto Alegre area. Participants were considered eligible to participate if they were male, right-handed, and between 19 and 40 years of age. They were screened for eligibility by phone to answer a structured questionnaire that assessed the following variables: sleep disorders, chronic diseases, substance abuse, or alcohol use in the past 6 months, use of psychotropic drugs, history of brain surgery, tumor, stroke or implantation of intracranial metal. Individuals responding affirmatively to any of these questions, those with contraindications for tDCS (Fregni et al., 2015) or if they presented a score on the Beck Depression Inventory (BDI) higher than 13 were excluded (Warmenhoven et al., 2012). We include males only to exclude the influence of the cyclical fluctuation of gonadal steroids during the menstrual cycle on pain measures (Stefani et al., 2012).

### Interventions

Remifentanil and saline (NaCl 0.9%) placebo solutions were prepared in coded (indistinguishable) infusion syringes attached to a continuous syringe pump with a dose of 0.06 μg⋅kg^-1^⋅min^-1^ (Gustorff et al., 2001). Except for T0, all other pain stimuli in the experiment had a concomitant infusion of remifentanil or saline.

**FIGURE 2:**
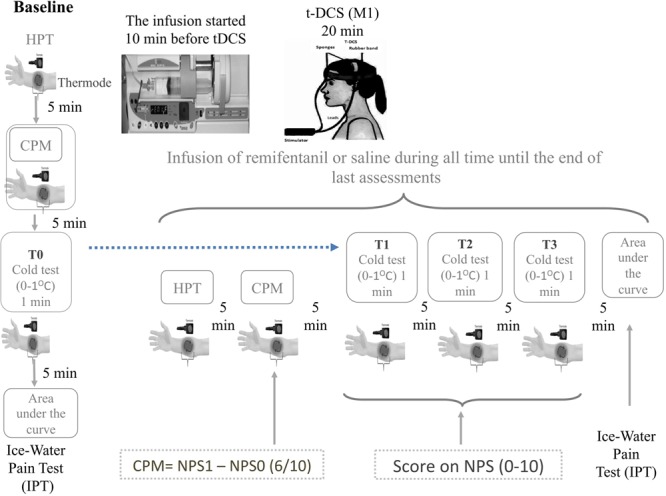
Experimental design. Timeline of each assessment pre and post intervention. The CPM is determined by the score on Numerical Pain Scale (NPST1) during the test stimulus (T1) minus de score on NPST0 during the conditioned pain stimulus (T0). The T0 is defined applying heat stimulus to provoke 6/10 in the NPS, which is determined individually before to apply the heterotopic stimulus. To determine the T1 a heat pain stimulus is applied in the dominant arm to provoke 6/10 in the NPS (0–10) when it is used simultaneously, a distant heterotopic nociceptive stimulus, generated by the immersion of non-dominant hand in cold water (zero to 1°C) for the 60 s. The area under the effect curve (AUEC) for reaction time (AUECVAS) is assessed during the ice-water pain test (IWPT), where the nociceptive stimuli were induced by the immersion of their hand in cold water (0 ± 1°C) for a maximum of 120 s. The repetitive cold test (rCOLDT) was induced by the immersion of their non-dominant hand in cold water (zero to 1°C) for the 60 s with a break of 5 min between each the trial.

The tDCS anode, active or sham, was positioned over the primary motor cortex (M1), and the cathode was placed on the right supraorbital region. tDCS was introduced 10 min after the beginning of infusion (remifentanil or saline) to ensure adequate concentration at the effector site according to pharmacokinetic characteristics of the drug. Each tDCS session lasted for 20 min. The rubber electrodes were inserted into a 35-cm^2^ sponge (moistened with NaCl). The current flow time and the intensity were set at 20 min and 2 mA, respectively. In sham-tDCS, the current flow was applied only for 30 s and turned off, to prevent the subjects from recognizing it from active tDCS. The timeline of study is presented in **Figure 2**.

### Outcomes

The primary outcome was the DPMS function as assessed by the NPS (0–10) during the QST applied simultaneously with a painful heterotopic stimulus, the rCOLDT and the change on the NPS (0–10) during the CPM-task. The secondary outcomes were the ascendant pain pathway and cortical involvement which was assessed by the HPT and the reaction time evaluated by the area under the curve using a Visual Analogue Scale (AUCVAS).

#### Primary Outcomes Assessment

##### CPM-task

To measure the CPM-task, we assessed the pain intensity during two tonic HPT test stimuli separated by a CPM-task. To induce a prolonged pain sensation to trigger CPM we used the HPT. The CPM-task consisted of immersion of the non-dominant hand in cold water (zero to 1°C) for 60 s. The QST procedure was introduced after 30 s of cold-water immersion over the right forearm (dominant forearm) using a temperature individually predetermined to produce a pain score of 6/10 in the NPS during the pretesting sessions. To determine the CPM, we used the difference between the pain score on the NPS (0–10) QST during cold-water immersion (QST+CPM) at the temperature of the point at which subjects felt 6/10 pain on the NPS scale [during the initial period (T0)].

##### Repetitive cold test (rCOLDT)

To examine the temporal summation of the second pain, we used a trial of three identical nociceptive stimuli to elicit a pain score of 6/10 in the NPS (test stimulus). A 5 min interval separated each trial. To produce a standard painful stimulus during each trial, they had to immerse their non-dominant hand in cold water (zero to 1°C) for 60-s. After 30-s of immersion, the test stimulus (QST) was applied, and they rated their pain on the NPS (0–10). The increase of 15% on the NPS (0–10) from the baseline was considered to present hyperalgesia. An accepted criterion to define a minimally important change on the NPS (0–10) is a modification of 10–20% (Dworkin et al., 2009). Thus, we assumed that scores on the NPS (0–10) during the rCOLDT that present an increase of at least 15% or more from the baseline indicated a presence of hyperalgesia (Dworkin et al., 2009). For the subsequent analysis, they were categorized into two groups, according to scores on the NPS (0–10): presence of hyperalgesia [increase of 15% or more in their pain score from the baseline (T0)] or absence of hyperalgesia [a change lower than 15% in the NPS (0–10) during the three trials (T1 to T3)].

#### Secondary Outcomes Assessment

##### Heat pain threshold (HPT)

The method of limits with a computer Peltier-based device thermode (30 mm × 30 mm) was used to assess the HPT (Schestatsky et al., 2011). The thermode was attached to the ventral aspect of the mid-forearm. The temperature was increased at a rate of 1°C/s, from 32°C to a maximum of 52°C. To determine the mean temperature of the HPT, we performed three assessments with an inter-stimuli interval of 40 s.

##### Ice-water pain test (IWPT)

The non-dominant hand was immersed in ice water (0 ± 1°C) for a maximum of 120 s. The subjects were asked to continuously score their maximal pain intensity perceived on a 0–10 cm electronic visual analog scale (VAS), where 0 represents ‘no pain’ and 10 represents the ‘worst pain imaginable.’ Subjects were recorded as 120 s if they did not withdraw their hand for the maximum time. For subsequent analysis of peak pain intensity, the area under the time curve was considered as well as the mean pain intensity. If a subject indicated 5 cm pain (i.e., midpoint between no pain and maximum pain) for 120 s, the AUEC value was 5 cm ^∗^ 120 s or 600 cm ^∗^ second. Higher values of the area under the effect curve (AUEC) for reaction time (AUECVAS) indicates longer reaction time response.

### Sample Size

The number of subjects in each study group was determined by the parameters of a previous survey (da Silva et al., 2015). To achieve 80% power at 1% significance, with a 0.5 variation coefficient, we need a total of 48 subjects divided into four equal groups (*n* = 12) in 1:1:1:1 ratio to test 1 point [*Standard deviation* (*SD* = 2)] mean a difference between groups for the NPS (0–10). The NPS (0–10) was used to assess both primary outcomes (rCOLDT and CPM-task).

### Randomization and Blinding

The sequence of randomization was generated by a computer with a fixed block size of 6. Forty-eight subjects were randomly allocated to receive treatment (a-tDCS/saline, s-tDCS/saline, a-tDCS/remifentanil, and s-tDCS/remifentanil). Before the recruitment phase, brown envelopes containing the protocol materials were prepared. Each envelope was sealed and numbered sequentially and contained an allocated treatment. Only the individuals responsible for administering the interventions were not blinded. All other participants were blinded to the allocated interventions.

#### Other Instruments and Assessments

Pain catastrophizing was assessed using the validated Brazilian-Pain Catastrophizing Scale (Sehn et al., 2012). Depression symptoms were screened using the BDI-II (Warmenhoven et al., 2012). We used the State-Trait Anxiety Inventory (STAI), adapted to Brazilian Portuguese, to measure the State-Trait Anxiety (Kaipper et al., 2010). The clinical assessment of sedation was determined by simultaneous recordings using a VAS for sleepiness (VASS 0–100) ranging from zero 0 (completely awake) to 100 (sleepiness). To assess safety, we used the Systematic Assessment for Treatment with a-tDCS questionnaire based on previously reported adverse events.

### Statistical Analyses

The differences between groups were examined with the analysis of variance (ANOVA) for parametric variables or the Kruskal–Wallis tests for non-parametric distributions. Categorical variables were examined using chi-square or Fisher’s exact and Kruskal–Wallis tests. The values are presented as the mean (standard deviation) or frequency. Continuous variables were tested for normality using the Shapiro–Wilks test.

A mixed ANCOVA model in which the independent variable was the time, the intervention (a-tDCS/saline, s-tDCS/saline, a-tDCS/remifentanil, and s-tDCS/remifentanil), interaction time vs. the treatment group, and subject identification was used to analyze the score change on the NPS (0–10) during the rCOLDT test and the change on the NPS (0–10) during the CPM-task.

A multivariate covariance analysis (MANCOVA) model was used to explore effects between the intervention groups in the multiple outcomes [Δ-AUECVAS and Δ-HPT, the Δ-value (post-intervention minus pre-intervention)]. The effect of all intervention groups on the outcomes were adjusted by sleepiness. All analyses were corrected for multiple comparisons using the Bonferroni test. All analyses were performed with two-tailed tests. We accepted a type I error of 5%. Statistical analyses were performed assuming intention-to-treat. The analyses were performed with the SPSS version 22.0 (SPSS, Chicago, IL, United States).

## Results

The characteristics were similar across the four groups as shown in **Table 1** (all *P*-values > 0.1). The incidence of tDCS associated side effects was reported by <15% of subjects and it was similar between groups. Itching in several body regions was reported in two subjects (16.6%) and four subjects (33.3%) in the a-tDCS/Remifentanil and s-tDCS/Remifentanil, respectively. Nausea was reported in four subjects (33.3%) and in five subjects (41.6%) in the a-tDCS/remifentanil and s-tDCS/saline, respectively. Vomiting was reported in one subject (8.3%) in the s-tDCS/remifentanil group. However, the frequency of these adverse effects was not statistically different between groups. The scores on the VAS (0–100) showed that a-tDCS/remifentanil and s-tDCS/remifentanil-induced higher sleepiness than the other groups (*P* < 0.01, for each comparison) (**Table 1**).

**Table 1:** Characteristics of the study sample.

	a-tDCS/remifentanil (*n* = 12)	s-tDCS/remifentanil (*n* = 12)	a-tDCS/saline (*n* = 12)	s-tDCS/saline (*n* = 12)	*P*
Age (years)	27.33 (5.08)	26.08 (2.67)	26.09 (3.41)	26.09 (3.41)	0.78
Education (years)	16.33 (4.68)	16.56 (4.67)	16.56 (4.65)	19 (1.3)	0.19
Weight (Kg)	77 (9.53)	73.62 (9.27)	71.5 (7.9)	75.3 (11.7)	0.45
State-anxiety	26.58 (2.34)	24. 0 (8.1)	20.33 (12.6)	20.33 (12.6)	0.53
Trait-anxiety	31.08 (2.6)	30.4 (10.3)	22.08 (7.72)	25.16 (15.5)	0.32
Depressive symptoms on the Beck Inventory	4.0 (5.1)	2.6 (3.2)	3.70 (3.45)	3.7 (3.45)	0.84
Pain Catastrophizing Scale	5.84 (6.82)	7.67 (9.15)	6.44 (7.78)	4.46 (7.48)	0.31
Cumulative mean of sleepiness score on VAS throughout the trials (0–100)	60 (15.22)^3,4^	57.91 (27.17)^3,4^	1.25 (3.16)^1,2^	13.41 (15.92)^1,2^	0.00

*Values are given as the mean (SD) or as a frequency according to the group (n = 48).*

### Treatment Effect on the Primary Outcome: NPS Score during the rCOLDT and the Change on NPS (0–10) during CPM-Task

Mean ± SD and median interquartile (Q25;75) of pain score on the NPS (0–10) during RCOLDT according to interventions at pre-intervention (T0), and post-intervention (T1 to T3) are presented in **Table 2**. The incidence of hyperalgesia was 22% in the group that received the a-tDCS/remifentanil compared to 8.3% in the group that received s-tDCS/placebo; the relative risk (RR) for the a-tDCS/remifentanil was 2.75 [confidence interval (CI) 95%, 1.26–5.88]. The incidence of hyperalgesia in the group that received the s-tDCS/remifentanil was 30.3% compared to s-tDCS/placebo, the RR for the s-tDCS/remifentanil was 3.87 [CI 95%, (1.87–8.01)]. The incidence of hyperalgesia in the group that received the a-tDCS/saline was 11% compared to s-tDCS/saline, the RR for the s-tDCS/remifentanil was 1.38 (CI 95%, 0.58–3.27). We observed that the RR to show r-IH increased approximately fourfold when the s-tDCS/remifentanil was used. Although the a-tDCS combined with remifentanil reduced the incidence of r-IH in 8%, the RR to induce hyperalgesia was yet significantly greater than those not receiving remifentanil.

**Table 2:** Pain score on NPS (0–10) during RCOLDT according to groups: Mean ± SD and median interquartile (*Q*_25;75_) pre-intervention (T0) and post-intervention (T1–T3).

Pain Score on NPS (0–10) during rCOLDT (primary)								
Time	a-tDCS/remifentanil		s-tDCS/remifentanil		a-tDCS/saline		s-tDCS/saline	
	*n* = 12		*n* = 12		*n* = 12		*n* = 12	
	Mean (*SD*)	Median (*Q*_25-75_)	Mean (*SD*)	Median (*Q*_25-75_)	Mean (*SD*)	Median (*Q*_25-75_)	Mean (*SD*)	Median (*Q*_25-75_)
T0	4.5 (1.56)	4 (2; 7)	4.33 (2.10)	4 (1; 8)	4.58 (1.75)	4 (3; 8)	4.5 (1.62)	4.5 (2; 7)
T1	3.92 (1.74)	3.5 (1; 7)	4.95 (2.05)	4 (1; 7)	4.5 (1.5)	5 (2; 7)	4.25 (1.48)	4.5 (2; 6)
T2	4.35 (2.09)	4 (1; 6)	5.45 (1.92)	5 (1; 7)	3.83 (1.34)	3.5 (2; 6)	3.5 (1.78)	3.5 (1; 7)
T3	4.20 (2.49)	4.5 (0; 6)	5.85 (2.45)	5 (1; 8)	3.4 (1.24)	3 (2; 6)	2.92 (1.92)	3.72 (0;7)
**OIH defined as an increase equal or higher than 15% on the NPS (0–10) from the T1 to T3**								
	22% (8/36)		31% (11/36)		11% (4/36)		8.33% (3/36)	

*OIH defined as an increase equal or higher than 15% on the NPS (0–10) from the T1 to T3 (n = 48).*

*The incidence was calculated considering the number of measures after receiving the intervention (T1–T3, that is three tests per each one of four interventions group, a total of 36 trials).*

**FIGURE 3:**
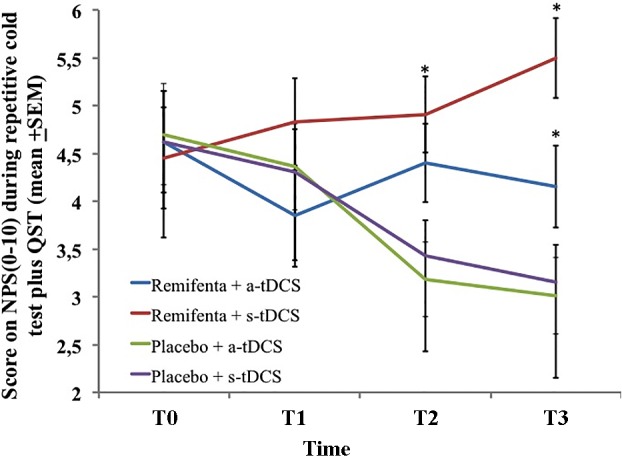
The change in NPS (0–10) during rCOLDT, at baseline before intervention and in three trials after tDCS and continuous infusion of the remifentanil or placebo in the four experimental groups. The error bars indicate standard error of the mean. The asterisk (^∗^) indicates differences between the remifentanil combined to a-tDCS or sham groups. All comparisons were performed by a mixed analysis of variance model, followed by the Bonferroni test for *post hoc* multiple comparisons. Numerical Pain Scale (NPS 0–10).

A mixed ANCOVA model in which the independent variable was the time, the intervention (a-tDCS/saline, s-tDCS/saline, a-tDCS/remifentanil, and s-tDCS/remifentanil), interaction time vs. the treatment group, and subject identification was used to analyze the score change on the NPS (0–10) during the rCOLDT test and the change on the NPS (0–10) during the CPM-task. The mean in the NPS (0–10) during the rCOLDT is presented in **Figure 3**. An ANCOVA mixed model revealed a significant main effect of interventions on the NPS (0–10) during the rCOLDT (*F* = 3.81; *P* = 0.01). The analysis showed a significant interaction between the group of interventions and time (*F* = 2.04; *P* = 0.04). The time effect was not observed (*F* = 1.43; *P* = 0.23). The effect of the sedation reported on the NPS (0–10) during the rCOLDT was not significant (*F* = 0.76; *P* = 0.38).

The change within the group was significant in all treatment groups (*P* < 0.001, for all comparisons). The mean (SD) on the NPS (0–10) during the rCOLDT pretreatment vs. the cumulative pain scores according to marginal means (T1–T3) in the a-tDCS/remifentanil was 4.62 (1.56) vs. 4.15 (1.55) and for the s-tDCS/remifentanil group was 4.45 (2.10) vs. 5.49 (1.04), respectively. Whereas, for the a-tDCS/saline, these values were 4.7 (1.72) vs. 3.11 (1.2) and for the s-tDCS/saline group these values were 4.62 (1.62) vs. 3.15 (1.62), respectively. The difference in the mean [CI 95%] within the a-tDCS/remifentanil group was 0.47 (CI 95%, 0.04 to 0.90), a small effect size (Cohen’s *f* = 20.3). The difference in the mean within the s-tDCS/remifentanil group was -1.04 (CI 95%, -1.63 to -0.44), a medium effect size (Cohen’s *f*2 = 0.49). Whereas in the a-tDCS/Saline, it was 1.59 (CI 95%, 1.10–2.08), a large effect size (Cohen’s *f*2 = 0.93) and in the s-tDCS/saline it was 1.47 (CI 95%, 1.01–1.93), a medium effect size (Cohen’s *f*2 = 0.72). It is possible to see that the effect size in the groups receiving remifentanil was determined by the increase in pain scores in the cumulative pain scores during the treatment, whereas the effect size observed when they are receiving a-tDCS or s-tDCS with saline was determined by a decrease in the pain scores during the treatment.

**FIGURE 4:**
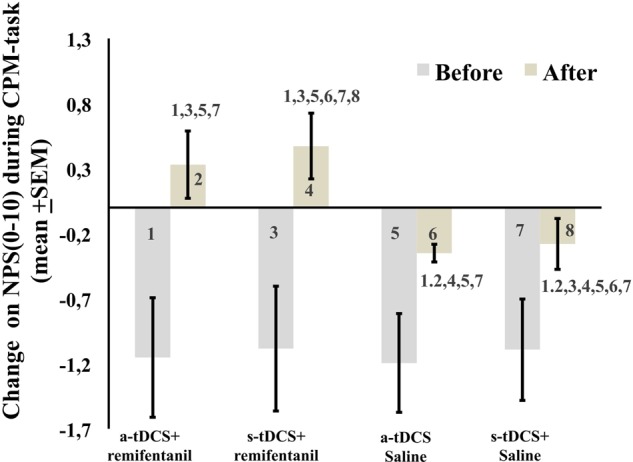
The change in NPS (0–10) during CPM-task, before intervention and immediately after in the four experimental groups. The error bars indicate standard error of the mean. Numbers show differences between four treatment groups. All comparisons were performed by a mixed analysis of variance model, followed by the Bonferroni test for *post hoc* multiple comparisons. Numerical Pain Scale (NPS 0–10).

The mean in the NPS (0–10) during the CPM-task is presented in **Figure 4**. An ANCOVA mixed model revealed a main effect of interventions on the CPM-task (*P* < 0.05) [*F* = (7.95; 3) = *P* < 0.001)]. Also, there is a significant effect of time (*F* = 17.01; *P* < 0.001) and a significant interaction between intervention and time *F* = (3.75; *P* < 0.001). The sleepiness level influenced the NPS during the CPM-task (*F* = 7.95, *P* < 0.001). The change on the NPS (0–10) during the CPM-task in groups receiving remifentanil showed positive values on the NPS scores, that is, it produced a disengagement of the DPMS.

### Treatment Effects on the Time Reaction HPT and the AUECVAS for Pain (Secondary Outcomes)

Patients receiving (a-tDCS/remifentanil), (s-tDCS/remifentanil), and (a-tDCS/saline) showed a greater HPT compared to (s-tDCS/saline). Their effect size assessed by the SMD within the (a-tDCS/remifentanil) group was 0.85 while the (s-tDCS/remifentanil) was 0.37. That is, the s-tDCS/remifentanil showed lower HPT compared to a-tDCS combined with the remifentanil or saline (**Table 2**). A MANCOVA analysis revealed a significant relationship between the intervention groups and the HPT as well as in the AUECVAS [Hotelling’s Trace = 0.78, *F*(9) = 3.41, *P* < 0.001]. The power of this analysis was 98%.

Sleepiness was inversely correlated with the response to the IPT assessed by the AUECVAS, in the sense that more sleepiness was associated with greater reaction time. The sleepiness had a medium effect size (Cohen’s *f*^2^ = 0.31) for the pain measured by AUECVAS (r-squared = 0.07, standard β coefficient = -2.72, *t* = -0.58, both *P* < 0.001). While the Sleepiness level had a small effect size for the HPT (Cohen’s *f*2 = 0.09) (r-squared = 0.07, standard β coefficient = 0.02, *t* = 2.16, both *P* < 0.001). The multivariate model is presented in **Table 3**.

**Table 3:** Treatment effect on HPT and AUECVAS between Groups: Mean ± SD, pre-intervention to post-intervention, mean difference with the confidence interval (95% CI) and standardized mean difference (SMD) (*n* = 48).

	Pre-intervention	Post-intervention	Mean difference (pre-intervention – post-intervention, 95% CI)	SMD
**Heat pain threshold (°C) (secondary)**				
a-tDCS/remifentanil	43.54 (3.13)	46.20 (2.80)	–2.66 (-1.35 to -3.97)^a^	0.85
s-tDCS/remifentanil	42.63 (1.95)	43.35 (2.04)	–0.72 (-0.35 to -1.79)^b^	0.37
a-tDCS/saline	43.44 (2.62)	45.17 (3.00)	–1.73 (-0.90 to -2.56)^a^	0.66
s-tDCS/saline	43.72 (2.55)	43.50 (2.32)	0.22 (-1.15 to 0.70)^c^	0.09
*P*-value^¥^	0.02			
**Ice-water pain test (IPT) [area-under-the-time-response curve (AUC)] (secondary)**				
a-tDCS/remifentanil	952.85 (139.06)	777.66 (223.65)	–175.19 (-284.18 to -66.20)^b,c^	1.26
s-tDCS/remifentanil	1036.43 (90.02)	802.88 (184.71)	–233.55 (-315.38 to -151.72)^b,c^	2.59
a-tDCS/saline	1020.10 (120.32)	1003.21 (127.54)	–16.89 (-68.80 to 35.02)^a^	0.15
s-tDCS/saline	1003.31 (84.15)	1005.33 (76.64)	2.03 (-24.45 to 28.50)^a^	0.03
*P*-value^¥^	0.02			

*R^*2*^ = 0.39. Repetitive cold test (RCOLDT).SD, standard deviation; CI, confidence interval; tDCS, transcranial direct current stimulation.Standardized mean difference (SMD) [(post minus pre)/baseline standard deviation the baseline standard deviation (SD) of sham tDCS/saline]. The effect size was interpreted as follows: small, 0.20; moderate, 0.50–0.60 and large, 0.80.*^¥^*Comparisons between the a-tDCS+remifentanil, s-tDCS+remifentanil, a-tDCS+saline analgesia, s-tDCS+saline analgesia groups performed using MANCOVA.Different superscripts (a, b, c) indicate significant difference among treatment groups after post hoc analysis adjusted by Bonferroni (P < 0.05).*

## Discussion

The main results of this study can be summarized by three major findings: (1) we showed that r-IH involves the dysfunction of the inhibitory pathways or up-regulation of the pain-facilitating pathways. This effect was demonstrated by the disengagement of the DPMS measured by the change in the NPS (0–10) during the CPM-task and by the summation effect on pain scores during the rCOLDT; (2) a-tDCS blocks r-IH. (3) a-tDCS/saline (as compared with s-tDCS/saline) has no effect on pain modulation during the rCOLDT in healthy subjects (likely due to a floor effect). Finally, according to the AUECVAS measurements, adjusted by the sleepiness, the remifentanil group showed a longer reaction time during the IPT compared to the other groups while the a-tDCS improved the HPT.

a-tDCS induced a large size effect to prevent r-IH, an effect with a statistical significance and further clinical relevance, especially because opioids are a powerful tool in the treatment of pain. Also, because OIH has been described with acute and chronic exposure, even using different types of opioids at high and low doses (Chen, 2014). Thereby, r-IH may be explained by a hyperexcitability of the dorsal horn neurons leading to an enhanced pain perception (Porreca et al., 2002). Also, according to previous reports, an increase of mμ-opioid receptor binding may decrease the ability to recruit endogenous opioids (Zubieta et al., 2003) and paradoxically increase the pain intensity or sensitivity (Lee et al., 2011). Another study observed that a high-dose of remifentanil decreased the mechanical hyperalgesia threshold and enhanced pain intensity (Lee et al., 2013). Additionally, previous studies provided convincing evidence that the mechanisms of OIH are a consequence of NMDA receptor activation, an increase of spinal excitatory neuropeptides induced by an increase of spinal dynorphins such as calcitonin gene-related peptide (CGRP) leading to a reduction in the reuptake of neurotransmitters that mediate nociception such as glutamate and substance P (Youssef et al., 2015).

The effect of tDCS mitigates part of the dysfunction in pain pathways induced by remifentanil as displayed by its effect on the NPS during the rCOLDT. This result fits nicely with the putative effects of tDCS in chronic neuropathic pain (Maarrawi et al., 2013). In this condition, it is believed that a lack of afferent stimuli may over-activate thalamic centers resulting in over processing of any sensory stimulus (Bolognini et al., 2013). Primary motor cortex stimulation during a-tDCS can re-establish this deafferentation partially. Our findings suggest that r-IH has a similar effect of blocking afferent sensory processing (inducing similar mechanisms of central sensitization), which a-tDCS seems to block. According to anatomical and electrophysiological data, the caudal medulla sub nucleus reticular dorsal (SRD) in the spinal bulb spinal loops are preferentially or exclusively activated by nociceptive stimuli (Wei et al., 2012). Hence, the responses of SRD neurons to noxious stimuli depend on the intensity and spatiotemporal features of the noxious stimuli, and in rodents, the SRD establishes reciprocal connections with the periaqueductal gray (Morgan et al., 1992). Thus, this is a plausible mechanism that may be involved in r-IH as observed in the current study, which we can indirectly assess.

Also, the r-IH observed in our study are aligned with the previous study, which measured the DNIC, in which exogenous opioids suppressed the endogenous opioid system and led to an increased sensitivity to pain (Ram et al., 2008). However, it is pertinent to emphasize that the greater pain score observed when individuals received remifentanil, without an anodal stimulation of M1, may be explained by a lack of the counteracting effects as described in the previous study using fMRI (Borsook and Becerra, 2006). This indicates that anodal stimulation of the M1 activates the endogenous opioid system (DosSantos et al., 2014). Apparently, another factor may be involved in this response, such as genetic polymorphism as reported in an experiment with healthy subjects after a single opioid administration (Jensen et al., 2009) and in a group of cancer patients (Rakvåg et al., 2005), which showed that after a single opioid administration, a repeated painful stimulus produced a hyperalgesic effect, which was associated with the COMT val158met polymorphism. The hypothesis of the involvement of the opioidergic system in tDCS is plausible if one considers that the endogenous opioid system is one important target of motor cortex stimulation to treat pain (Maarrawi, 2007). Accordingly, previous studies showed that the density of opioid receptors predicted pain relief when the motor cortex is stimulated either in those suffering chronic or acute postoperative pain (Maarrawi et al., 2013). Similarly, after a single session of anodal M1-tDCS a decreased binding of the selective μ-opioid receptor agonist [11-C] Carfentanil in pain-related regions (e.g., thalamus, precuneus, PAG, prefrontal cortex and the anterior cingulate cortex) was observed in healthy subjects (DosSantos et al., 2014) as well as in the injured subjects (DosSantos et al., 2012). Therefore, the present results also indicate the contribution of the endogenous opioid system in the analgesic effects induced by M1-a-tDCS.

The central role of the glutamatergic system to induce OIH may be due to an increase of glutamate available to NMDA receptors by a decreased reuptake of glutamate (Lee et al., 2011). However, in the human, this effect can be assessed only indirectly by behavioral and psychophysical measures. Although the neurobiological processes involved in the effect of a-tDCS to modulate hyperalgesia induced by remifentanil is not clear, we can at least propose that there is an improvement in the dysfunction of the DPMS with tDCS treatment. Even though this is a matter of intense debate, an antidromic modulation of thalamocortical pathways occurs when the motor cortex is stimulated, which plays a central role in the analgesia induced by M1 to decrease pain-induced thalamic hyperactivity (Tsubokawa et al., 1993). However, further studies are necessary to establish the clinical relevance for tDCS counteracting hyperalgesia induced by remifentanil.

It is important to address several issues concerning the design of our study: First, we include males only to exclude the influence of the cyclical fluctuation of gonadal steroids during the menstrual cycle on pain threshold in females (Smith et al., 2002). Second, when opioids are applied, the blinding can be troublesome due to their side effects. Sedation might mainly affect the pain scores and the reaction-time. These effects can explain the higher rate of individuals guessing the intervention correctly when they received remifentanil compared to subjects who did not. However, sedation is an intrinsic property of opioids. Thus, its effect was considered in the analysis to control its possible confounding effect. Thereby, it is improbable that unblinding changes the directions of our conclusions. Third, using this experimental design to induce sensitization, the RR of remifentanil induced hyperalgesia increased significantly, similarly to the incidence reported in previous studies run in clinical settings (Fletcher and Martinez, 2014). Thereby, these results enforce the theory that remifentanil can induce hyperalgesia and the a-tDCS can partially block this paradoxical effect. Fourth, it is important to take into account that we used a cutoff point in the percentage of increase NPS (0–10) to define hyperalgesia based on a clinical criteria to define a clinical effect with a minimal relevance (Dworkin et al., 2009). Fourth, tDCS is a low-cost technique with a low incidence of minor adverse effects. It is easy to apply and it is an efficient technical solution to conduct blinded studies of both the patients and experimenters (Gandiga et al., 2006). Finally, the greater reaction time in the groups that received remifentanil compared to the others that not received remifentanil can be explained by the sedation analgesia effect of opioids. However, in the groups s-tDCS/remifentanil and s-tDCS/saline, a lower HPT was observed compared to the groups that received a-tDCS, either combined with remifentanil or saline. In fact, this result is in agreement with previous studies which showed that a-tDCS improved the pain threshold (Vaseghi et al., 2015). On the other hand, the current results are in disagreement with previous studies that showed a higher sensitivity in the pain perception by the QST, either in patients managed with long-term opioid therapy (Compton et al., 2001) or after acute opioid dependence in non-addicted humans (Compton et al., 2003). A possible reason to explain these differences between our findings and previous studies is the type of opioids, dose and sample characteristics.

## Conclusion

These findings suggest that effects of a-tDCS prevent the summation response induced by r-IH during rCOLDT, and also that a-tDCS blocked the disengagement of DPMS. Thereby, they revealed that tDCS could be a new approach at contra-regulating paradoxical mechanisms involved in r-IH. In fact, these results have physiological implications to support an understanding of the mechanisms involved in the pathophysiology of r-IH as well as supporting the development of new approaches to treat OIH.

## Author Contributions

GB and FF conceived the study, participated in its design and coordination, performed the statistical analysis, and helped for drafting the manuscript. SP, FL, and AS participated in the sequence alignment. LS and AP conceived the study, participated in its design and coordination, and helped for drafting the manuscript. IT participated in the sequence alignment and drafted the manuscript. WC is responsible for maintaining the study records, conceived the study, participated in its design and coordination, helped for drafting the manuscript, and performed the statistical analysis.

## Conflict of Interest Statement

The authors declare that the research was conducted in the absence of any commercial or financial relationships that could be construed as a potential conflict of interest.
